# Hepatitis C Virus Nonstructural 3/4A Protein Dampens Inflammation and Contributes to Slow Fibrosis Progression during Chronic Fibrosis In Vivo

**DOI:** 10.1371/journal.pone.0128466

**Published:** 2015-06-01

**Authors:** Ruchi Bansal, Lars Frelin, Erwin Daniel Brenndörfer, Gert Storm, Jai Prakash, Matti Sällberg

**Affiliations:** 1 Department of Laboratory Medicine, Division of Clinical Microbiology, Karolinska Institutet, Stockholm, Sweden; 2 Targeted Therapeutics, Department of Biomaterials, Science and Technology, MIRA Institute for Biomedical Technology and Technical Medicine, Faculty of Science and Technology, University of Twente, Enschede, The Netherlands; 3 Department of Pharmaceutics, Utrecht Institute for Pharmaceutical Sciences, Faculty of Science, Utrecht University, Utrecht, The Netherlands; University of Navarra School of Medicine and Center for Applied Medical Research (CIMA), SPAIN

## Abstract

HCV infection typically induces liver injury and inflammation, which appears to be responsible for the associated fibrogenesis. To date, the mechanism underlying the different rates of disease progression remains unclear. The aim of the study is to understand the possible role of the HCV non-structural (NS) 3/4A protein in the fibrosis progression. We used NS3/4A-expressing transgenic mice (NS3/4A-Tg) to accomplish the goals of the study. Different stages of liver fibrosis were induced in wild-type and NS3/4A-Tg mice by single carbon tetrachloride (acute) or multiple injections for 4 (intermediate) or 8 (chronic) weeks. Fibrotic parameters, inflammatory responses and hepatocyte turnover were extensively examined. Hepatic expression of HCV NS3/4A did not induce spontaneous liver damage. However, NS3/4A expression exerted contrasting effects during acute and chronic liver damage. During early fibrogenesis and intermediate fibrosis (4 weeks), NS3/4A-Tg mice exhibited enhanced liver damage whereas reduced fibrosis was observed in NS3/4A-Tg during chronic liver fibrosis (8 weeks). Furthermore, attenuated inflammation was observed in NS3/4A-Tg during chronic fibrosis with increase in M2 macrophages, hepatocyte proliferation, decreased hepatocyte apoptosis and decreased ductular reaction. In conclusion, during early fibrogenesis, HCV NS3/4A contributes to liver damage. While, during chronic liver fibrosis, NS3/4A dampens inflammation and induces hepatocyte regeneration thereby contributing to slow fibrosis progression to promote its survival or persistence.

## Introduction

Hepatitis C virus (HCV) is a major cause of chronic hepatitis with increasing morbidity and mortality. An estimated 3% of the world population-more than 170 million people worldwide are infected with hepatitis C virus [1,2]. Liver disease attributable to HCV currently represents the main indication for liver transplantation [3]. Progression to chronic Hepatitis C occurs in most people acutely infected (55% to 86%) with HCV, and persistent infection is an important cause of cirrhosis, end-stage liver disease and hepatocellular carcinoma [4].

The natural history of chronic HCV infection is insidious in most patients. Although the disease is mild and stable or slowly progressive in about 70% of chronically infected patients, the remaining 30% develop progressive liver disease culminating in cirrhosis and hepatocellular carcinoma [5,6]. Progression to liver cirrhosis usually takes 20–40 years but in some patients severe fibrosis can develop rapidly leading to death within 5–10 years from the onset of infection [7]. The mechanisms responsible for different rates of fibrosis progression are largely unknown but likely reflect differences in the interplay between virus and host immune response. A number of factors have been shown to influence the fibrosis progression e.g alcohol consumption, age and gender [8]. The role of viral proteins and key cell types hepatic stellate cells (HSCs) and macrophages in fibrosis progression has not been investigated yet.

HCV genome encodes for three structural proteins (core C, glycoproteins E1 and E2) and seven non-structural (NS) proteins (P7, NS2, NS3, NS4A, NS4B, NS5A and NS5B), which coordinates the intra-cellular processes of the viral life cycle. HCV NS3/4A is a multifunctional complex composed of NS3 and its cofactor NS4A. It harbours serine protease and NTPase/RNA helicase activities and is therefore essential for viral polyprotein processing, RNA replication and viral assembly [9,10]. Apart from participating in the viral life cycle, HCV NS3/4A also facilitates viral persistence by interfering with intrahepatic signal transduction. In particular, it has been shown that NS3/4A mediates immune-modulatory effects by triggering NFκB activation and TNFα production [11]. This has been shown to prevent hepatocyte apoptosis and to support liver regeneration thereby promoting viral persistence [11]. But how these activities are linked to fibrosis progression is still unknown.

HCV is a non-cytopathic virus, largely infecting hepatocytes and exploiting its cellular machinery for replication and survival [12]. The underlying mechanisms of HCV-related fibrosis, involvement of HCV proteins in the process of fibrogenesis and importantly, the contribution of non-parenchymal cells i.e. HSCs and macrophages to this process remains largely obscure.

In this study, we aim to understand the involvement of HCV NS3/4A and key cell types (hepatic stellate cells and macrophages) in the progression of fibrosis. To accomplish the goals of the study, different stages of fibrosis (acute, intermediate and chronic) were induced in NS3/4A-transgenic (Tg) and wild-type mice following treatment with carbon tetrachloride (CCl_4_). Thereafter, fibrotic parameters, inflammatory response (macrophages) and hepatocyte turnover (proliferation and apoptosis) were examined. We found that during the acute and intermediate phase of fibrosis, NS3/4A contributed to increased fibrosis progression with increased stellate cells activation. No difference in inflammation, hepatocyte turnover and ductular reaction was observed in NS3/4A-Tg in comparison to wild-type mice. In contrast, during chronic liver injury: (a) slow fibrosis progression, (b) dampened inflammation with increased number of M2 phenotypic macrophages, and (c) increased hepatocyte proliferation and decreased apoptosis were observed. In conclusion, this study clearly demonstrate the role of HCV NS3/4A in inflammation, fibrosis progression, viral persistence and liver regeneration.

## Materials and Methods

### Ethics statement

All the animal experiments in this study were performed in strict accordance with the guidelines and regulations for the Care and Use of Laboratory Animals, by the ethical committee at Karolinska Institutet, Sweden. The experimental procedures performed in this study have been approved and the mice were monitored according to the guidelines issued by the ethics committee.

### Mice

CBA x C57BL/6 mice transgenic (Tg) for full-length NS3/4A with functional protease (HCV genotype 1a) [13] were bred and housed at the animal facility at Karolinska Institutet, Division of Comparative Medicine, Clinical Research Centre. All the transgenic animals were analysed for the presence of genomic NS3/4A transgene as described earlier [13]. The respective wild-type (WT) mice were purchased from Charles River Laboratories (Sulzfeld, Germany).

### CCl_4_-induced acute liver injury mouse model

Male CBA x C57BL/6 mice (WT and NS3/4A-Tg; 6–10 weeks old; n = 5 per group) were treated with a single intra-peritoneal injection of olive oil or CCl_4_ (1ml/kg in olive oil) at day 0. At day 2 (48hrs) all mice were sacrificed; blood and livers were collected for subsequent analysis.

### CCl_4_-induced liver fibrosis mouse models

Male (WT and NS3/4A-Tg; 6–10 weeks old; n = 5 per group) were treated with olive oil or increasing doses of CCl_4_ (week 1: 0.5ml/kg; week 2: 0.8ml/kg and week 3–8: 1ml/kg prepared in olive oil) twice weekly by intra-peritoneal injections for 4 or 8 weeks as previously described. The mice (WT and NS3/4A-Tg) were sacrificed at week 4 and week 8 by cervical dislocation. Blood and livers were collected for subsequent measurements.

### Histology and immunohistochemistry

Liver tissues were harvested and transferred to Tissue-Tek OCT embedding medium (Sakura Finetek, Torrance, CA), and snap-frozen in iso-pentane chilled in dry ice. Cryosections (4μm) were cut using a Leica CM 3050 cryostat (Leica Microsystems, Nussloch, Germany). The sections were air-dried and fixed with acetone for 10 min. Tissue sections were rehydrated with PBS and incubated with the primary antibody in appropriate dilution (refer to **S1 Table**) for 1hr at room temperature. Sections were incubated with horseradish peroxidase (HRP)-conjugated secondary antibody for 30 min at room temperature. Then, sections were washed with 1x PBS for 5 min. Following incubation with HRP-conjugated tertiary antibody for 30 min, sections were then washed thrice with 1x PBS. Thereafter, peroxidase activity was developed using AEC (3-amino-9-ethyl carbazole) substrate kit (Life Technologies, Gaithersburg, Md) for 20 min and nuclei were counterstained with hematoxylin (Fluka Chemie, Buchs, Switzerland). Cells or sections were mounted with VectaMount AQ medium (Vector Laboratories, Burlingame, CA). The staining was visualized and the images were captured using light microscopy (Nikon eclipse E600 microscope, Nikon, Tokyo, Japan).

### Quantitative histochemical analysis

For quantitative histological analyses, stained sections were scanned at high resolution using Hamamatsu NanoZoomer Digital slide scanner 2.0 HT (Hamamatsu Photonics, Bridgewater NJ). Images were viewed using NanoZoomer Digital Pathology (NDP) viewer software (Hamamatsu Photonics). Images (100x) covering the entire section (from NDP) were imported into NIH ImageJ software (NIH, Bethesda, MD) and were analyzed quantitatively at a fixed threshold.

### Quantitative real-time PCR

Total RNA from liver tissues was isolated using SV total RNA isolation system (Promega Corporation, WI, USA) according to the manufacturer’s instructions. The RNA concentration was quantitated by a UV spectrophotometer (NanoDrop Technologies, Wilmington, DE). Total RNA (1μg) was reverse transcribed in a volume of 20μl using iScript cDNA Synthesis Kit (Bio-Rad, Hercules, CA). All the primers were purchased from Sigma-Genosys (Haverhill, UK). Real-time PCR was performed using the 2x SensiMix SYBR and Fluorescein Kit (Bioline Inc., QT615-05, Luckenwalde Germany), 20ng cDNA and pre-tested gene-specific primer sets (listed in **S2 Table)**. The cycling conditions for the BioRad CFX384 Real-Time PCR Detection System were 95°C for 10 min, 40 cycles of 95°C/15 sec, 58°C/15 sec and 72°C/15 sec. Finally, RNA transcription levels were determined by C_T_ values (C_T_ >35 rejected) and relative quantities were calculated by the ΔΔC_T_. Transcripts were normalized with GAPDH (for mouse) as a house-keeping gene.

### Statistical analyses

All the data are presented as mean ± standard error of the mean (SEM). The graphs and statistical analyses were performed using GraphPad Prism version 5.02 (GraphPad Prism Software, Inc., La Jolla, CA, USA). Multiple comparisons between different groups were performed by one-way analysis of variance (ANOVA) with Bonferroni post-test. The differences were considered statistically significant at p < 0.05.

## Results

### NS3/4A increased fibrogenesis during acute liver injury

Acute liver injury was induced in HCV NS3/4A-Tg and wild-type control mice **(S1 Fig, panel A)**, and expression of extracellular matrix (ECM) proteins (collagen I) and HSC markers (α-SMA and Desmin) was determined. As can be seen in the microscopic images, hepatic expression of NS3/4A did not induce spontaneous liver damage. Following exposure to CCl_4_, NS3/4A-Tg mice showed increased fibrogenesis as depicted in S1 Fig, panel B. This suggests that following liver damage, intrahepatic expression of NS3/4A potentiates liver fibrogenesis accompanied by increased stellate cell activation and proliferation.

### NS3/4A induces fibrosis at the intermediate stage but slow down the process in chronic fibrosis

**Fig 1:**
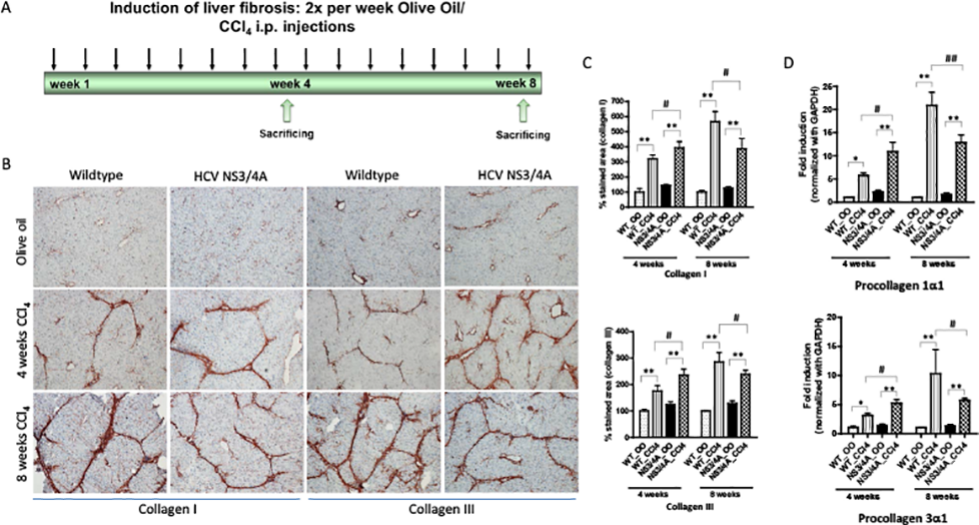
Analysis of fibrosis in wild-type and NS3/4A-Tg mice after CCl_4_ administration. **(A)** Regimen of liver fibrosis induction (sacrificed after 4-weeks (intermediate fibrosis) and 8-weeks (chronic fibrosis) of CCl_**4**_). **(B)** Representative photomicrographs and **(C)** quantitative analysis of collagen-I and collagen-III stained liver sections of 4-weeks and 8-weeks olive-oil treated and CCl_**4**_-treated wild-type and NS/4A-Tg mice. Scale bars, 200μm. (D) mRNA expression of procollagen-1α1 and -3α1 (normalized with GAPDH) in the livers of wild-type and NS3/4A-Tg. For quantitative and mRNA analysis, groups were normalized to olive-oil treated wild-type mice. Bars represent mean ± SEM of n = 5. *p<0.05; **p<0.01; #p<0.05.

Since it has been shown that HCV infected patients have varied degree of fibrosis, we investigated if HCV NS3/4A influences fibrosis progression at different stages of fibrosis. We therefore administered CCl_4_ in wild-type and HCV NS3/4A-Tg mice for 4 weeks (intermediate fibrosis) and 8 weeks (chronic fibrosis) (**Fig 1A**). We then examined the ECM deposition by immunohistochemistry and quantitative PCR at the different fibrotic stages.

During 4 weeks of fibrosis, we observed significant up-regulation of collagen I and III protein and mRNA expression (p<0.05), as also evidenced by increased collagen bridges in NS3/4A-Tg as compared to wild-type CCl_4_-treated control mice (**Fig 1B–1D**). After 8 weeks of CCl_4_ administration, there was a significant increase in collagen expression in CCl_4_-treated wild-type mice while this increase was blunted in NS3/4A-Tg during chronic liver injury. No significant differences were observed in cytochrome P450 (**S2 Fig**) and TGFβ expression levels (**S3 Fig**) in wild-type and NS3/4A-Tg at different stages of fibrosis. Overall, these results suggest HCV NS3/4A slows down the fibrogenic process at the chronic stage of fibrosis.

### Effect of HCV NS3/4A on hepatic stellate cells at different stages of fibrosis

**Fig 2:**
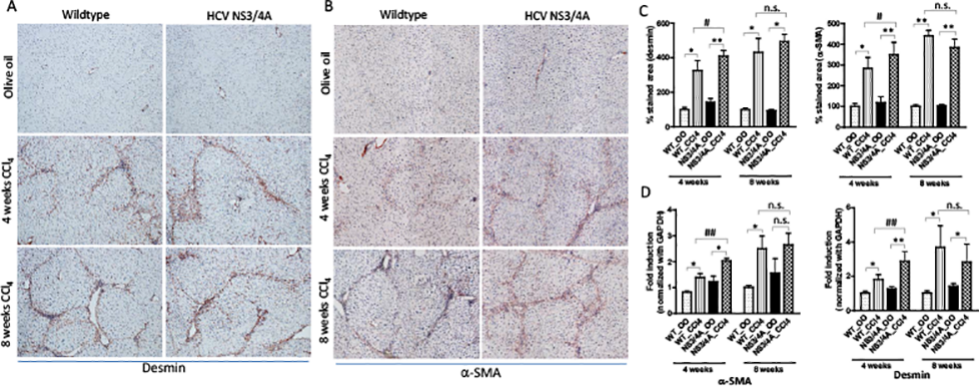
Hepatic stellate cells activation and proliferation in the livers of wild-type and NS3/4A-Tg mice after CCl_4_ administration. Representative pictures of **(A)** desmin- and **(B)** α-SMA-stained liver sections of 4-weeks and 8-weeks olive-oil treated and CCl_**4**_-treated wild-type and NS/4A-Tg mice. Scale bars, 200μm (**C**) quantitative analysis of desmin- and α-SMA stained liver sections. (D) mRNA expression of desmin and α-SMA (normalized with GAPDH) in the livers of wild-type and NS3/4A-Tg. For quantitative histological and mRNA analysis, groups were normalized to olive-oil treated wild-type mice. Bars represent mean ± SEM of n = 5 per group. *p<0.05; **p<0.01; #p<0.05; ##p<0.01; n.s. denotes non-significant.

To understand the role of HSCs in HCV-induced fibrogenesis, we studied different HSCs markers in wild-type and NS3/4A-Tg mice at intermediate and chronic stage of fibrosis. As observed earlier, increased fibrosis (or ECM deposition) at intermediate stage correlated with the significant increase in HSCs activation and proliferation in NS3/4A-Tg and wild-type mice treated with CCl_4_ (**Fig 2**). The increase in HSC markers was more pronounced in NS3/4A-Tg as compared to wild-type mice (**Fig 2**). In contrast, no significant differences in HSC activation and proliferation were observed in wild-type and NS3/4A-Tg mice during chronic liver fibrosis (**Fig 2**) indicating that the differences in collagen deposition between wild-type and NS3/4A-Tg mice were not due to stellate cells. This suggests the involvement of other factors (or cells) during this fibrotic stage leading to slow progression of fibrosis in NS3/4A-Tg.

### Decreased fibrosis in the presence of NS3/4A expression during chronic stage correlated with dampened inflammation and an increase in M2 macrophage

**Fig 3:**
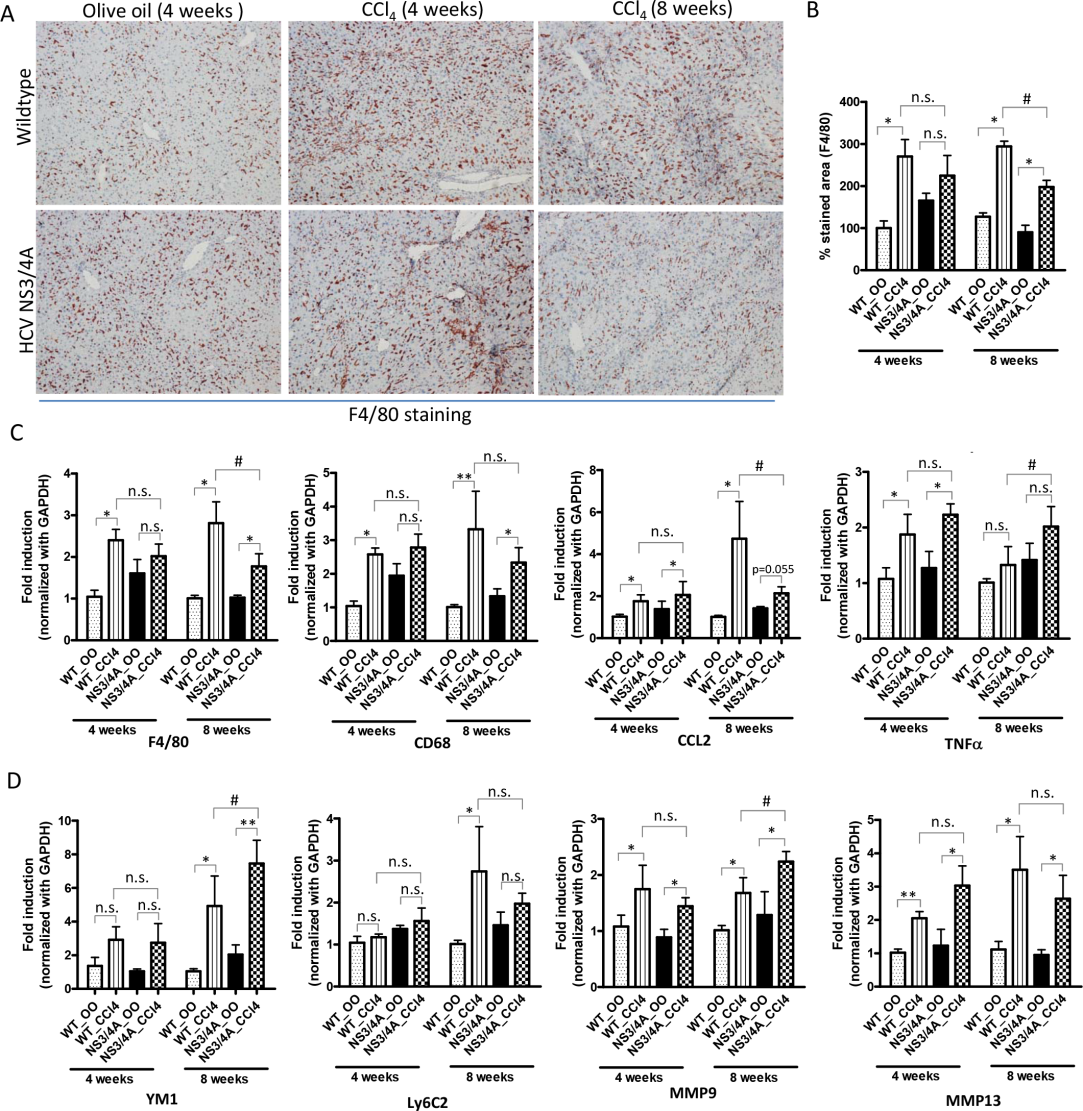
Inflammation in the livers of wild-type and NS3/4A-Tg mice after CCl_4_ administration. **(A)** Representative pictures and **(B)** quantitative histological analysis of F4/80-stained liver sections of 4-weeks and 8-weeks olive-oil treated and CCl_4_-treated wild-type and NS3/4A-Tg mice. Scale bars, 200μm. Quantitative PCR analysis of **(C)** inflammatory parameters (F4/80, CD68, CCl2 and TNFα), **(D)** M1 macrophage-specific (Ly6C2) and M2 macrophage-specific (YM1), and MMP-9 and -13 in the livers of wild-type and NS3/4A-Tg. For quantitative histological and PCR analysis, groups were normalized to olive-oil treated wild-type mice. Bars represent mean ± SEM of n = 5 per group. *p<0.05; **p<0.01; #p<0.05; n.s denotes non-significant.

We examined the role of NS3/4A expression in liver inflammation and in macrophage plasticity and polarization during different stages of fibrosis. Following F4/80 immunostaining, we observed a significant increase in the number of macrophages in wild-type mice and tendency of increased macrophage numbers in NS3/4A-Tg after 4 weeks of CCl_4_ administration, which was similar at mRNA expression levels (F4/80 and CD68) (**Fig 3A–3C**). Furthermore, elevated expression of pro-inflammatory cytokines MCP1 (CCL2) and TNFα was observed in wild-type and NS3/4A-Tg following 4 weeks of fibrosis (**Fig 3C**). In contrast, during the chronic phase of fibrosis, dampened inflammation was evidenced in NS3/4A-Tg as compared to wild-type mice as shown i.e. by a reduced number of macrophages and attenuated levels of CCL2 (**Fig 3A–3D**).

With respect to macrophage plasticity and polarization, during chronic infection, NS3/4A-Tg induced M2 macrophage polarization as seen by significantly up-regulated YM1 (M2 marker) expression and down-regulation of Ly6C2 (M1 marker) expression (**Fig 3D**). In addition, both wild-type and NS3/4A-Tg mice were characterized by increased expression of MMP9 and MMP13, which was more pronounced in NS3/4A-Tg (particularly MMP9) (**Fig 3D**) favouring an increase in collagen degradation at chronic stage of fibrosis. Furthermore, we have performed quantitative gene expression analysis for IFNγ (M1-stimulating cytokine) and IL-13 (M2-stimulating cytokine) that showed lowered IFNγ mRNA levels and significantly higher levels of IL-13 in the livers of NS3/4A-Tg versus wild-type mice during chronic liver injury, indicating suppressive M1 response and increased M2 response respectively (**S3 Fig**). Taken together, these results suggests that HCV NS3/4A contributes to dampened inflammation and M2 macrophage polarization thereby resulting in attenuated or slow fibrosis progression.

### HCV NS3/4A increases hepatocyte proliferation and reduced hepatocyte apoptosis during chronic fibrosis

**Fig 4:**
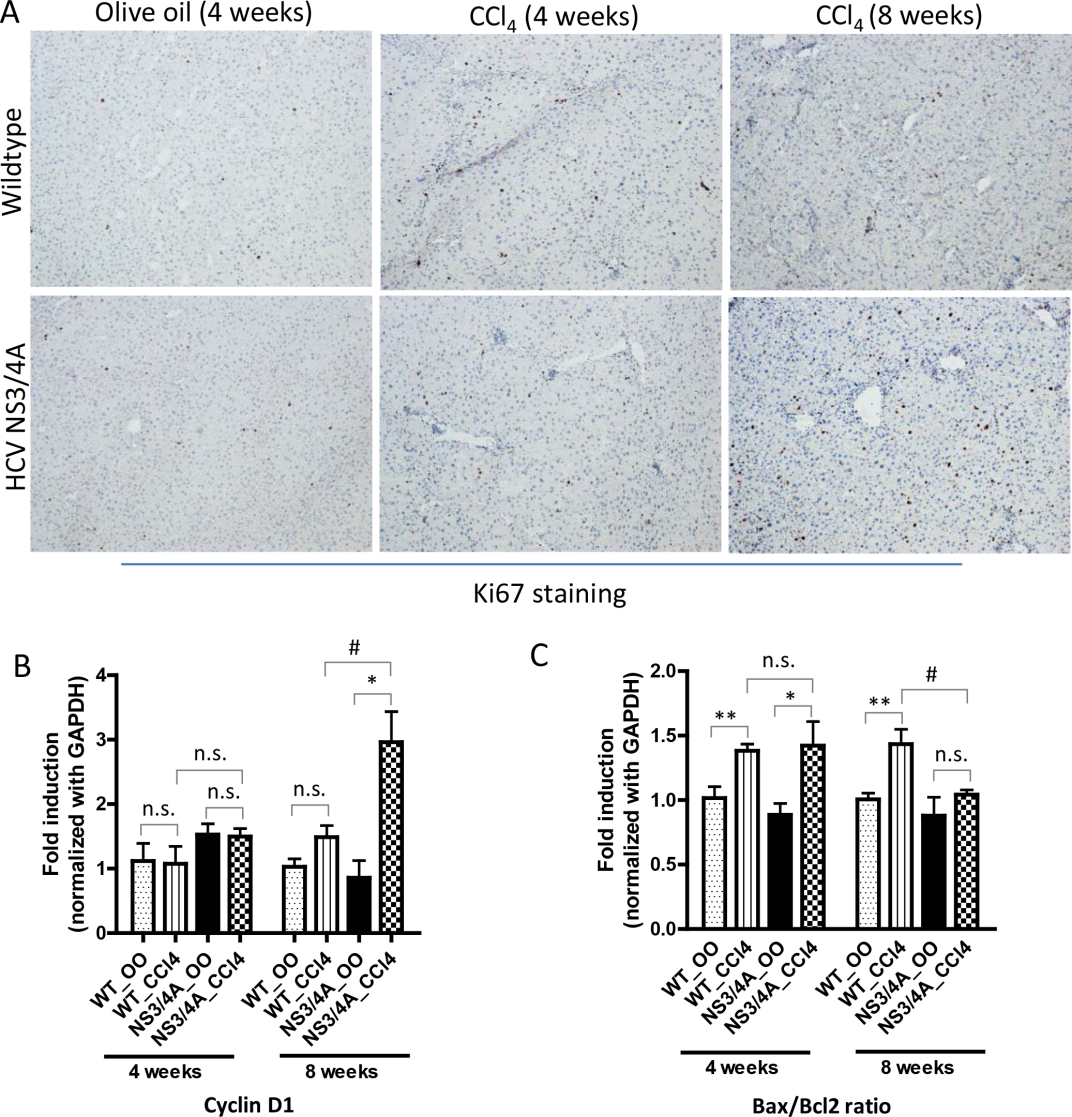
Hepatocyte proliferation and apoptosis in wild-type and NS3/4A-Tg mice after CCl_4_ administration. **(A)** Representative pictures of Ki67-stained liver sections of 4-weeks and 8-weeks olive-oil treated and CCl_**4**_-treated wild-type and NS3/4A-Tg mice. Scale bars, 200μm. Quantitative real-time PCR analysis (normalized with GAPDH) of **(B)** cyclin D1 and **(C)** Bax/Bcl2 ratio in the livers of wild-type and NS3/4A-Tg. For quantitative PCR analysis, groups were normalized to olive-oil treated wild-type mice. Bars represent mean ± SEM of n = 5 per group. *p<0.05; **p<0.01; #p<0.05; n.s. denotes non-significant.

Since apoptotic hepatocytes contributes to increased fibrosis and hepatic fibrosis has also been associated with hepatocyte replicative arrest, we next examined the hepatocyte proliferation and apoptosis in wild-type and NS3/4A-Tg mice at different stages of fibrosis. Hepatocyte proliferation was examined using Ki67 immunohistochemistry and cyclin D1 gene expression levels. Low expression of Ki67 was observed in control mice suggesting basal renewal of the hepatocyte pool in the liver parenchyma. Hepatocyte proliferation was slightly but not significantly increased after 4 weeks of CCl_4_ administration, which was similar in both wild-type and NS3/4A-Tg mice (**Fig 4A**).

During chronic fibrosis however, a significant increase in Ki67-positive hepatocytes was observed in NS3/4A-Tg in comparison to wild-type mice (**Fig 4A**). This increase was further confirmed by significant up-regulation in cyclin D1 transcripts in NS3/4A-Tg compared to wild-type mice (**Fig 4B**). Further, increased hepatocyte proliferation was paralleled by reduced apoptosis of hepatocytes as evidenced by significantly decreased Bax/Bcl2 ratio in NS3/4A-Tg as compared to wild-type mice (**Fig 4C**).

We performed mRNA expression analysis for NS3/4A gene in 4 weeks and 8 weeks olive oil or CCl_4_-treated transgenic mice. We found that following 4 or 8 weeks of olive oil or CCl_4_ administration did not affect NS3/4A gene expression. Furthermore, NS3/4A mRNA expression was increased in 8 weeks, most probably due to increased hepatocyte proliferation and reduced hepatocyte apoptosis (**S5 Fig**). These results suggest that NS3/4A promotes hepatocyte regeneration and exerts anti-apoptotic effects (also in part by causing increase in hepatocyte-protective TNFα (**Fig 3C**) to promote viral infection and persistence.

### Decreased hepatic fibrosis was associated with decreased ductular reaction in NS3/4A transgenics

**Fig 5:**
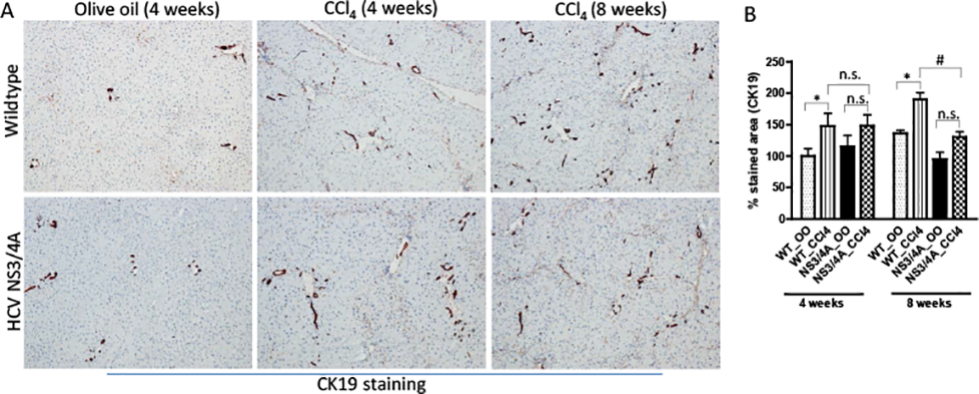
Analysis of ductular reaction in the livers of wild-type and NS3/4A-Tg mice after CCl_4_ administration. **(A)** Representative pictures and **(B)** quantitative histological analysis of CK19-stained liver sections of 4-weeks and 8-weeks olive-oil treated and CCl_**4**_-treated wild-type and NS3/4A-Tg mice. Scale bars, 200μm. For quantitative histological analysis, groups were normalized to olive-oil treated wild-type mice. Bars represent mean ± SEM of n = 5 per group. *p<0.05; #p<0.05; n.s. denotes non-significant.

Since hepatocyte proliferation is linked to ductular reaction and liver fibrogenesis during HCV infection; we examined the ductular reaction in wild-type and NS3/4A-Tg by CK19 immunostaining. CK19 staining was confined to bile duct cholangiocytes and 4 weeks CCl_4_ treatment resulted in a significant increase in CK19-positive cells in both wild-type and NS3/4A-Tg mice. However, no differences between NS3/4A-Tg and wild-type mice could be observed (**Fig 5A and 5B**). In contrast, during chronic CCl_4_ administration, increased CK19-positive biliary epithelial cells were observed in wild-type animals as compared to NS3/4A-Tg mice (**Fig 5A and 5B**). This suggests that the increase in fibrosis progression after 8 weeks CCl_4_ treatment seen in wild-type mice is concomitant with enhanced ductular reaction. However, the reduced fibrosis in NS3/4A-Tg during chronic stage of fibrosis is complemented by a decreased ductular reaction.

## Discussion

In this study, we have for the first time, demonstrated a role of the Hepatitis C virus NS3/4A complex in fibrosis progression. We have shown that during induction of acute and intermediate fibrosis, NS3/4A enhances fibrosis progression while at chronic stage of fibrosis, NS3/4A dampens inflammation, slows down fibrosis progression and promotes hepatocyte regeneration thereby providing permissive environment that supports viral persistence.

Several *in vitro* studies have suggested that HCV proteins can directly promote fibrogenesis. For example, a study with an *in vitro* co-culture system of primary rat stellate cells with HCV core-expressing HepG2 cells suggested the contribution of HCV proteins (HCV core, NS5A and HCV subgenomic replicon) in hepatic fibrogenesis via production of profibrotic factors and activation of HSCs [14,15]. Furthermore, HCV viral proteins, such as core, NS3 and NS5A, have been reported to alter hepatocyte metabolism and mitochondrial dysregulation resulting in reactive oxygen radicals (ROS) production, thereby leading to the induction of TGFβ and oxidative stress both *in vitro* and *in vivo* [16–19]. More recently, it has been shown that hepatocytic expression of HCV proteins contributes to increased fibrosis in presence of CCl_4_ owing to enhanced ductular reaction, hepatocyte growth/replicative arrest and enhanced ROS production [20].

Our findings suggest that NS3/4A protein expression does not induce spontaneous liver fibrosis in mice. Nevertheless, we demonstrate that NS3/4A, in part, determines the degree of fibrosis depending on the stage of fibrosis. Whereas during acute and intermediate fibrosis, NS3/4A protein expression potentiates fibrosis, while at later chronic stage, it slows down the fibrosis progression. These effects are relevant since HCV infection may lead to mild or stable disease in ‘slow progressors’ or progress rapidly to cirrhosis or liver related death in ‘rapid progressors’ [21]. Factors leading to this differential progression of fibrosis are still unknown. Recently, Farci etal., 2012 has shown that disease severity in HCV patients is predicted by viral evolutionary dynamics (adaptive immunity) and MCP1 (CCL2) levels [21]. The fibrosis progression is mainly determined by the activation status of HSCs, but in our study collagen deposition during chronic fibrosis did not correlate with HSC activation signifying the role of other important factors in this phenomenon.

It is well known that liver inflammation e.g. macrophages are important determinants/regulators of fibrosis [22]. In our study, we found that during initial liver damage, accelerated fibrogenesis is secondary to sustained intrahepatic inflammation leading to iterative hepatocyte injury. In contrast during chronic fibrosis, hepatocytic expression of NS3/4A led to the significant reduction in liver inflammation owing to reduced inflammatory cells infiltration resulting in the decreased production of chemokines e.g. CCL2 and macrophage polarization to the M2 suppressive phenotype. It has also been reported earlier that hepatocytic NS3/4A expression modulates intrahepatic immunity. Particularly type I/II dendritic cells which have phenotypic similarity with type-I IFNγ-producing plasmacytoid dendritic cells was found to be significantly reduced in NS3/4A-Tg livers [13] suggesting reduced IFNγ production thereby suppressing M1 macrophage polarization. More recently, Brenndorfer etal., 2014, have shown that NS3/4A-Tg mice have lowered hepatic CCR5 ligand, CCL3 and increased IL-10 levels as compared to wild-type mice [23] favouring M2 macrophage polarization. In addition, we have also shown here that during chronic liver injury, NS3/4A-Tg have decreased IFNγ mRNA levels and significantly higher levels of IL-13 indicating increased M2 response and suppressive M1 response. As also shown earlier [11], NS3/4A expression in hepatocytes during LPS-induced liver damage led to increased hepatocyte regeneration and decreased apoptosis most likely via the NFκB signalling pathway suggesting a role of the NS3/4A protein in viral persistence.

A strong relationship between ductular reaction and hepatic fibrosis severity has been reported in both rodents and humans [24–26]. We also found that at the late stage of fibrosis, decreased hepatic fibrosis was correlated with decreased ductular reaction.

In conclusion, we have demonstrated that HCV NS3/4A protein partially determines the severity of liver fibrosis at different stages of HCV infection by modulating inflammation and hepatocyte regeneration. At early stage of fibrosis, NS3/4A promotes fibrosis, HSC activation, liver inflammation and hepatocyte apoptosis while at the chronic phase, this in turn slows down fibrosis progression, dampens inflammation, prevents hepatocyte apoptosis and promotes hepatocyte regeneration thereby providing permissive environment for its survival and persistence suggesting that the use of NS3/4A inhibitors in HCV therapy may exert effects beyond the viral life cycle.

## Supporting Information

S1 Fig Immuno-histochemical analysis of fibrosis parameters in wild-type and NS3/4A-Tg mice following CCl_4_-induced acute liver injury. Induction of acute liver injury (48hrs) by CCl_4_ administration (A). Representative photomicrographs of collagen I-, α-SMA- and Desmin-stained liver sections of olive-oil treated and CCl_4_-treated wild-type and NS/4A-Tg mice (B). Scale bars, 100μm.
(TIF)

S2 Fig Cytochrome P450 levels in wild-type and NS3/4A-Tg following CCl_4_ administration. mRNA expression analysis of Cytochrome P450 E1 (normalized with GAPDH) in the livers of wild-type and NS3/4A-Tg mice. Bars represent mean ± SEM of n = 5 per group. *p<0.05; n.s. denotes non-significant.
(TIF)

S3 Fig TGFβ gene expression levels in wild-type and NS3/4A-Tg following CCl_4_ administration. mRNA expression analysis of TGFβ (normalized with GAPDH) in the livers of wild-type and NS3/4A-Tg mice. Bars represent mean ± SEM of n = 5 per group. *p<0.05; n.s. denotes non-significant.
(TIF)

S4 Fig IFNγ and IL-13 mRNA expression levels in wild-type and NS3/4A-Tg following CCl_4_ administration. mRNA expression analysis of IFNγ and IL-13 (normalized with GAPDH) in the livers of wild-type and NS3/4A-Tg mice. Bars represent mean ± SEM of n = 5 per group. *p<0.05, #p<0.05; n.s. denotes non-significant.
(TIF)

S5 Fig NS3/4A mRNA expression levels in wild-type and NS3/4A-Tg following CCl_4_ administration. mRNA expression of NS3/4A (normalized with GAPDH) in the livers of wild-type and NS3/4A-Tg mice. Bars represent mean ± SEM of n = 5 per group. Wild-type (olive oil or CCl_4_-treated) mice showed C_T_ values above the threshold (non-detectable levels). n.s. denotes non-significant.
(TIF)

S1 Table Primary and secondary antibodies used for the immunohistological stainings. (PDF)

S2 Table Sequence of the primers used for the quantitative real-time PCR. (PDF)
