# Supplementary material for: Hepatitis C Virus Nonstructural 3/4A Protein Dampens Inflammation and Contributes to Slow Fibrosis Progression during Chronic Fibrosis In Vivo
Source: PLoS One. 2015 Jun 1;10(6):e0128466. doi: 10.1371/journal.pone.0128466 (PMC4450868; doi:10.1371/journal.pone.0128466)
Supplement: S1 Table — (PDF) [file pone.0128466.s006.pdf]

S1 Table: Primary and secondary antibodies used for the immunohistological stainings

Primary Antibody	Source	Dilution
Polyclonal rabbit α-SMA	Abcam	1:100
Polyclonal goat anti-collagen I	Southern Biotech	1:100
Polyclonal goat anti-collagen III	Southern Biotech	1:100
Polyclonal rabbit Cytokeratin 19	Novus Biologicals	1:100
Polyclonal goat anti-desmin	Santa Cruz	1:100
Polyclonal rat anti-F4/80 (MCA497)	AbD Serotec	1:500
Polyclonal rabbit anti-Ki67	Millipore	1:100
Secondary Antibody	Source	Dilution
Polyclonal Goat anti-rabbit IgG	DAKO	1:100
Polyclonal Rabbit anti-rat IgG	DAKO	1:100
Polyclonal Rabbit anti-Goat IgG	DAKO	1:100
