# Supplementary material for: Hepatitis C Virus Nonstructural 3/4A Protein Dampens Inflammation and Contributes to Slow Fibrosis Progression during Chronic Fibrosis In Vivo
Source: PLoS One. 2015 Jun 1;10(6):e0128466. doi: 10.1371/journal.pone.0128466 (PMC4450868; doi:10.1371/journal.pone.0128466)
Supplement: S2 Table — (PDF) [file pone.0128466.s007.pdf]

S2 Table: Sequence of the primers used for the quantitative real-time PCR

Gene	Forward primer sequence (5'-3')	Reverse primer sequence (5'-3')
Bax	CTCAAGGCCCTGTGCACTAA	CACGGAGGAAGTCCAGTGTC
Bcl2	CTTTGAGTTCGGTGGGGTCA	AGTTCCACAAAGGCATCCCA
CD68	TTCTGCTGTGGAAATGCAAG	AGAGGGGCTGGTAGGTTGAT
Cyclin D1	CCATGCTCAAGACGGAGGAG	ATCCAGGTGGCCACGATTTT
CYP2E1	AAGAATGAGTTCTCTGGCCG	CCAGTCACGGAGGATACTTA
Desmin	ATGCAGCCACTCTAGCTCGT	CTCATACTGAGCCCGGATGT
F4/80 (EMR1)	TGCATCTAGCAATGGACAGC	GCCTTCTGGATCCATTTGAA
GAPDH	ACAGTCCATGCCATCACTGC	GATCCACGACGGACACATTG
IFNγ	AGCAAGGCGAAAAAGGATGC	TCATTGAATGCTTGGCGCTG
IL-1β	GCCAAGACAGGTCGCTCAGGG	CCCCCACACGTTGACAGCTAGG
IL-13	GTATGGAGTGTGGACCTGGC	CTCTGGGTCCTGTAGATGGC
Ly6C2	TGGACAGTACTCACGCTACA	GGCACTCCATAGCACTCGTA
MCP1 (CCL2)	GTGCTGACCCCAAGAAGGAA	GTGCTGAAGACCTTAGGGCA
MMP13	CCAGAACTTCCCAACCATGT	GTCTTCCCCGTGTTCTCAAA
MMP9	CGTCGTGATCCCCACTTACT	AACACACAGGGTTTGCCTTC
NS3/4A	GTTGTCGTCGTGGCAACTG	GAGGAAGCGTGGTTGTCTCA
Procollagen 1α1	TGACTGGAAGAGCGGAGAGT	ATCCATCGGTCATGCTCTCT
Procollagen 3α1	ACTGTCCCACGTAAGCACTG	CAGGAGGGCCATAGCTGAAC
TGFβ	GGACTCTCCACCTGCAAGAC	CTGGCGAGCCTTAGTTTGGA
TNFα	AGGCTGCCCCGACTACGTGC	CAGCGCTGAGTTGGTCCCCC
YM1 or CHI3L3	ACTTTGATGGCCTCAACCTG	AATGATTCCTGCTCCTGTGG
α-sma	ACTACTGCCGAGCGTGAGAT	CCAATGAAAGATGGCTGGAA

Abbreviations: α-sma, alpha smooth muscle actin; Bax, Bcl2-associated X protein; Bcl2, B-cell CLL/lymphoma 2; MCP1 (CCL2), macrophage chemotactic protein 1 (chemokine C – C motif ligand 2); CD68, cluster of differentiation 68; F4/80 or EMR1, EGF-like module-containing mucin-like hormone receptor-like 1; GAPDH, Gylceraldehyde 3-phosphate dehydrogenase; IL-1β, Interleukin 1 beta; Ly6C2, mouse lymphocyte antigen 6 complex, locus C2; MMP9 or MMP13, Matrix metalloproteinase 9 or 13; TNFα, tumor necrosis factor alpha; YM1 or CHI3L3, chitinase 3-like-3; CYP2E1, Cytochrome P450 E1; TGFβ, Transforming growth factor; NS3/4A, Nonstructural protein 3/4A; IL-13, Interleukin-13; IFNγ, Interferon gamma.
